# Depression and its associated factors among people who use drugs attending the Methadone Treatment Clinic at Tumbi Hospital in Pwani, Tanzania: A cross-sectional study

**DOI:** 10.1371/journal.pmen.0000446

**Published:** 2025-10-28

**Authors:** Anna Chrispo Charle, Masunga K. Iseselo, Sofia Sanga

**Affiliations:** 1 Department of Internal Medicine, Tumbi Regional Hospital, Pwani, Tanzania; 2 Department of Clinical Nursing, Muhimbili University of Health and Allied Sciences, Dar es Salaam, Tanzania; Northern Ontario School of Medicine: NOSM University, CANADA

## Abstract

Opioid use disorder is a major global health concern. While methadone treatment is effective in treating opioid use disorder, individuals often experience severe mental health issues, particularly depression. This study aimed to assess the prevalence of depression and its associated factors among clients attending a methadone clinic in the Pwani Region, Tanzania. A cross-sectional study was conducted at Tumbi Hospital to assess the prevalence of depression and its associated factors among 261 clients at the methadone treatment clinic. Participants were recruited through simple random sampling. Depression-related data were collected using the Patient Health Questionnaire-9 (PHQ-9). Descriptive statistics were used to analyze the socio-demographic and clinical data and prevalence of depression. Furthermore, bivariate and multivariate logistic regression were used to determine the factors associated with depression. A P-value of less than 0.05 with a 95% confidence interval was used to identify the statistically significant association. The mean age of respondents was 40.28 years (SD ± 8.29), and 98.1% were male. The prevalence of depression was 73.9%, with 47.9% of respondents experiencing minimal depression. Females had a 15 times higher risk of depression (adOR = 15.126; P = 0.021) compared to their counterparts. Clients with a history of incarceration were at twice the higher risk of depression (adOR = 2.371; P = 0.012), while not using tramadol for pain management had a reduced risk of depression (adOR = 0.853; P < 0.001). Additionally, clients with a lack of social support had a five times increased risk of depression (adOR = 4.606; P = 0.028). The high prevalence of depression among individuals in methadone treatment underscores the need for comprehensive mental health interventions, with a focus on addressing gender disparities, post-incarceration support, and substance use monitoring.

## Introduction

Substance use disorders (SUDs) are a significant global health concern, profoundly impacting individuals, families, and entire communities [1]. The widespread use and misuse of drugs continues to rise, with 292 million people worldwide affected [2]. Opioid use, in particular, remains a critical issue, leading to devastating health outcomes and high mortality rates [2]. A substantial area of concern revolves around the use of opioids, affecting an estimated 13.5 million individuals worldwide. Among these individuals, 9.2 million have developed a specific dependence on heroin. A distinctive pattern of substance use statistics in Sub-Saharan Africa has come to light, suggesting the presence of a unique subset of individuals with SUDs. For example, an estimated 18,000–30,000 individuals in Kenya are engaged in injecting drug use [3]. This data underscores the existence of a specific category of individuals with SUDs within the country. In Tanzania, estimates indicate that Tanzania is home to a population of individuals who inject drugs (PWIDs) ranging from 25,000–50,000 [4]. This is a huge number of individuals who use opioids that need treatment intervention.

Methadone treatment has emerged as a vital intervention in addressing opioid use disorders (OUDs), offering individuals a structured path to recovery [5]. It is a first-line medication for OUD, reduces all-cause and overdose mortality [6], increases treatment engagement, and prevents harm related to injection drug use, including HIV and hepatitis C [7,8]. In Tanzania, a publicly funded methadone treatment clinic was established in 2011 at the Muhimbili National Hospital (MNH) in Dar es Salaam [9]. Since then, methadone for OUD has been available to outpatients through opioid treatment programmes (OTPs). The Drug Control and Enforcement Authority (DCEA) licenses and certifies OTPs and sets detailed requirements for clinic characteristics, patient eligibility, dosing standards, observed dosing, and counselling [10]. Currently, there are 12 methadone treatment clinics nationwide, including three in Dar es Salaam. The clinics offer methadone treatment and various other pharmacological, behavioural, and psychosocial support services, such as tuberculosis testing and treatment, clinical medical services, psychotherapy, and case management through on-site social workers at no cost [11]. Despite these considerable advantages, there is a persistent presence of depressive symptoms in some patients who use these methadone treatment clinics [12], highlighting a challenging issue that warrants investigation.

Numerous studies have investigated the relationship between substance use and mental health disorders, with a particular focus on OUDs and depression [13,14]. Research has shown that individuals undergoing methadone treatment are at a heightened risk of experiencing depressive symptoms, which can hinder their recovery and overall well-being [12]. While global data on methadone treatment attendance and its outcomes are available [15], there is a notable gap in region-specific research, particularly in Sub-Saharan Africa. In this region, unique patterns of substance use and mental health issues exist, yet detailed data on methadone attendance and the prevalence of depression among people who use are limited. Previous studies have highlighted the need for targeted interventions and a better understanding of the factors contributing to depression in this population [14–16].

The co-occurrence of SUDs and other mental health issues must be examined to understand how these conditions influence depression predictors and treatment outcomes. This is because the influence of peer dynamics and the effectiveness of psychosocial interventions for OUDs are key components of this inquiry, as reported in other studies [17,18]. However, much remains to be explored regarding the specific predictors of depression within the context of methadone treatment, particularly in low-resource settings. Collectively, three broad areas that include access to service, co-occurrence of SUDs and other mental health issues, and influence of peer dynamics address the complex web of depression predictors within the unique context of methadone treatment. This study aimed to determine the prevalence and determinants of depression among individuals undergoing methadone treatment in Tanzania. The findings from this research inform healthcare providers and policymakers about the need for comprehensive mental health support in methadone treatment programmes, ultimately improving the well-being of individuals struggling with both substance use and depression.

## Methodology

### Study design

This was a cross-sectional study to assess the prevalence and determinants of depression among people who use drugs attending methadone treatment. This design was appropriate to our study population, as we wanted to obtain the factors associated with depression at a given time to align with objectives [19]. According to various sources, the design was used to provide critical insights into the mental health status of this population during the study period [20].

### Settings

The study was conducted at the methadone clinic in Tumbi Regional Referral Hospital in Pwani, Tanzania. The estimated number of people who use and inject drugs was 25% [21]. Tumbi Hospital provided both outpatient and inpatient care services and had 254 beds. According to the hospital report for August 2023, out of 476 registered methadone clinic clients, 312 were currently undergoing treatment per month. The eligibility criteria for the methadone treatment programme include current physical opiate dependence and addiction; objective findings and subjective reports of the patient that support the need for pharmacotherapy; a judgement that the patient is capable of understanding and participating in a treatment programme; and an expressed willingness of the patient to enter treatment after the nature of that treatment has been carefully explained to him or her [22]. The clinic offered various services, including tuberculosis screening and treatment with Daily Observed Treatment (DOT), hepatitis screening, HIV testing and treatment with DOT, and pre-exposure prophylaxis for people who inject drugs [11]. Additionally, services for physical and psychological conditions were offered. This clinic served as the primary healthcare facility for individuals seeking treatment for substance use disorders.

### Study population and eligibility criteria

The participants of this study were individuals 18 years and above who had a confirmed diagnosis of OUDs and were seeking treatment. The diagnosis was made after the client fulfilled the following criteria: 1) opioid dependence, 2) evidence of recent drug injection, and 3) positive opiate urine screening [22]. The study participants were currently receiving methadone as part of their OUD treatment plan. However, individuals with severe cognitive impairments or neurological conditions and severe psychiatric comorbidities such as schizophrenia, bipolar disorder, or other severe mental illnesses were excluded. This might have affected their ability to provide informed consent or accurately respond to interview or survey questions.

### Sample size and sampling procedure

A simple random sampling technique was employed. In this method, a sampling frame was created, compiling a list of all eligible individuals to attend the clinic on a particular day. Each client in the clinic was assigned a sequential number ranging from 1 to 476 based on the order of their attendance. The sample size was estimated based on a study in Iran that showed a 49% prevalence of depression among opioid users in the outpatient clinic [23]. Assuming an alpha value of 5%, a sample size of 288 participants was estimated using the Kish-Lisle formula of cross-sectional studies [24]. After obtaining the randomly chosen respondents, they were monitored based on the days they came to take their medication and were approached willingly to participate in the study, considering that substance users often miss doses. Each day a computer number generator was used to select participants. They were assigned numbers, and a computer system generator was used to select participants. The first author, who had experience working with people who use drugs, was responsible for the organisation and recruitment of the respondents at the study site.

### Data collection method and tool

Data collection for this study was facilitated using a structured questionnaire to determine the social demographic and clinical characteristic factors associated with depression among substance users attending the methadone treatment clinic through an interviewer-administered structured questionnaire. Which was developed in English and then translated into the Swahili language.

#### Standard structured questionnaire.

An interviewer-administered questionnaire was used to collect the data. The questionnaire assessed sociodemographic data such as age, gender, marital status, education level, and employment status. Also, details on health and treatment, such as substance use, the duration and pattern of heroin use, methadone dosage, and physical health status, were elicited. Questions like “Have you experienced any physical health problems or disabilities as a result of substance use?” were asked. This question was used to assess if the respondent had acquired any injury, HIV or Hepatitis B Virus (HBV) due to injecting drug behaviour. No laboratory investigation or clinical records were requested to confirm the responses. This data provided insights into the respondents’ clinical characteristics and their experiences with substance use and treatment.

#### Patient Health Questionnaire (PHQ-9).

For this study, we used the Swahili version of the PHQ-9, a 9-item instrument developed by Kroenke et al. [25] to screen for depression. This was a structured tool that can be used to screen for and diagnose depressive disorders, depending on the clinical setting. Respondents indicated the frequency of depression symptoms over the past 2 weeks on a 4-point scale, ranging from 0 (never) to 3 (nearly every day). The total score ranged from 0 to 27, with higher scores indicating a higher likelihood of major depressive disorder, with a cut-off point of 9. The participants with a score of 9 and above were considered to have depression, and those with scores less than 9 had no depression. Generally, the total scores of 5, 10, 15, and 20 represented the cut-off points for mild, moderate, moderately severe, and severe depression, respectively. Item 9 was a question that screens for suicide risk. The respondents who answered “yes” to question 9 were referred for immediate assistance to mental health providers for further mental health support at the clinic. The tool had been validated in Tanzania and previously used for assessing depression in various populations, with a Cronbach’s alpha of 0.89 [26].

### Data collection procedure

Data collection took place from the 2nd to the 25th day of May 2024. The data were collected in collaboration with the researcher and two research assistants, registered nurses from the methadone clinic who were hired and provided with a two-day training session before the start of data collection. The researcher provides comprehensive training to the research assistants on the data collection procedures, ethical considerations, and how to handle any issues that may arise during the process. They were also trained to understand the study objectives.

In this study, the researcher and research assistants interviewed the respondents and filled out the questions following the instructions provided. Research assistants facilitated the process, assisting respondents as needed, and ensuring that all completed questionnaires were collected and stored securely. All data were obtained from the questionnaire. In addition to interviewing and filling out the questionnaire, the researcher oversaw the entire process, ensuring ethical compliance and data quality control. On average, each questionnaire took approximately 10 minutes to complete filling it.

### Data processing and analysis

The dataset was first inspected to understand its structure and identify any obvious issues, such as missing values. A Statistical Package for Social Science (SPSS) version 27 was used to analyse the data. Using descriptive statistics and visual inspection tools (e.g., spreadsheets), duplicate entries and any identified duplicate rows were removed to ensure each respondent’s data was only represented once. We also formatted to rule out the inconsistencies by performing cross-checking variables (e.g., age and date of birth). Data transformation enabled statistical tests, such as encoding categorical variables and converting categorical data into numerical format, to allow the use of statistics. Descriptive statistical data were organised and analysed using univariate analysis and presented through tables using means, frequencies, standard deviations, and ranges for each variable to be studied. Bivariate analyses were performed to determine the association of social demographic and clinical characteristics with depression. Multiple logistic regression was used to identify independently associated risk factors for all bivariate variables with associations at p-value < 0.20 to adjust for confounders. A p-value of 0.05 and below was considered statistically significant.

### Ethical considerations

Ethical clearance was obtained from the Institutional Review Board at the Muhimbili University of Health and Allied Sciences with Reference No. DA 282/298/01.C/2199, and permission to collect data was secured from Tumbi Hospital management. Informed consent was obtained from respondents, ensuring they understood the study’s objectives, procedures, and risks clearly before recruitment. The respondents were encouraged to participate voluntarily, highlighting that there were no consequences for opting out of participation. Confidentiality was maintained, and steps were taken to minimise risks, with prompt referrals to healthcare services for distressed respondents. Respondents were told there were no tokens to participate in the study, but the findings obtained will help improve health care, especially for patients attending methadone clinics.

## Results

### Socio-demographic characteristics of respondents

A total of 261 participated in the study, which is 91% of the required sample size. The respondent’s mean age was 40.28 with a standard deviation (SD) of ±8.29 years. The majority, 98.1% (n = 256), were male. About half 48.3% (n = 126) of the respondents were between the ages of 35 and 44 years, and the majority 63.2% (n = 165) of them were single. In terms of education, 70.9% (n = 185) had primary education. Employment status showed that 83.9% (n = 219) were business owners. Regarding incarceration history, 60.1% (n = 157) had never been incarcerated (**Table 1).**

**Table 1:** Social demographic characteristics (N = 261).

Variable	Category	Frequency	Percent (%)
**Age (in years)**	18-24	4	1.5
	25-34	59	22.6
	35-44	**126**	**48.3**
	45 and above	72	27.6
**Gender**	Male	**256**	**98.1**
	Female	5	1.9
**Marital status**	single	**165**	**63.2**
	married	66	25.3
	Divorced	30	11.5
**Level of education**	Primary	**185**	**70.9**
	Secondary	51	19.5
	Tertiary	7	2.7
	Illiteracy	18	6.9
**Employment status**	Employed	17	6.5
	Unemployed	25	9.6
	Business owner	**219**	**83.9**
**Incarceration**	Never	**157**	**60.1**
	Once	79	30.3
	More than once	25	9.6

### Clinical characteristics of respondents

Regarding the duration in the programme, 40.2% (n = 105) of respondents had been in a methadone clinic for more than 24 months. Most of the respondents, 99.6% (n = 260), visited the clinic more than 6 days a week. A large proportion, 81.2% (n = 212), reported having no physical health problems or disability as a result of substance use. Most respondents, 73.6% (n = 192), had used painkillers without a prescription. Of those who used painkillers, 68.8% (n = 134) used diclopar (a medicine used to relieve pain and inflammation). However, 86.2% (n = 225) reported to have used substances in the last 30 days. Of those who used substances, 97.8% (n = 220) used tobacco, while the majority, 63.1% (n = 178), had stopped using alcohol. Support during stress was common, reported by 77.0% (n = 201) of respondents, with 62.8% (n = 164) turning to family for support. Sharing information during times of stress was common, with 80.5% (n = 210) of individuals. Additionally, the family was the main source of support for sharing joy or sorrow for 59.0% (n = 154) of individuals. A vast majority, 93.1% (n = 243), felt loved or important, mainly by family (76.2%, n = 199). Additionally, 85.8% (n = 224) did not have a family history of depression or mental illness (**Table 2).**

**Table 2:** Clinical characteristics of respondents (N = 261).

Variable	Category	Frequency	Percent (%)
**Duration in the methadone program**	3 month-9Month	60	23.0
	10 Months- 16 Months	62	23.8
	17 Months-23Months	34	13.0
	More than 24Months	**105**	**40.2**
**Frequency of visiting methadone clinic per week**	5 days	1	0.4
	More than 6 days	**260**	**99.6**
**Reported physical health problems or disability as a result of substance use**	Injuries	11	4.2
	HIV	8	3.1
	HBV	6	2.3
	TB	24	9.2
	None	**212**	**81.2**
**Use pain killer without prescription as a substance**	Yes	**192**	**73.6**
	No	69	26.4
**Use of diclopar without prescription as a substance**	Yes	**134**	**68.8**
	No	58	30.2
**Use tramadol without a prescription as a substance**	Yes	65	33.9
	No	**127**	**66.1**
**Use morphine/Heroine without a prescription**	Yes	9	4.7
	No	**183**	**95.3**
**Diazepam without prescription as a substance**	Yes	73	38.0
	No	**119**	**62.0**
**Used substances in the last 30 days**	Yes	**225**	**86.2**
	No	36	13.8
**Use Tobacco**	Yes	**220**	**97.8**
	No	5	2.2
**Use of alcohol**	Yes	83	36.9
	No	**142**	**63.1**
**Use of cannabis**	Yes	53	23.6
	No	**172**	**76.4**
**Support during stress**	Yes	**201**	**77.0**
	No	60	23.0
**A person to share information during stress**	Family	**164**	**62.8**
	Friends	23	8.8
	Other special person	19	7.3
	None	55	21.1
**Sharing** **information with person**	Yes	**210**	**80.5**
	No	51	19.5
**Person to share with joy or sorrow**	Family	**154**	**59.0**
	Friends	34	13.0
	Anyone special	26	10.0
	none	47	18.0
**The feeling of ever loved/being important**	Yes	**243**	**93.1**
	No	18	6.9
**Being loved by**	Family	**199**	**76.2**
	Friends	36	13.8
	Special	10	3.8
	none	16	6.1
**Family history of depression or mental illness**	Yes	37	14.2
	No	**224**	**85.8**

### Prevalence of depression among clients attending methadone clinics

**Fig 1:**
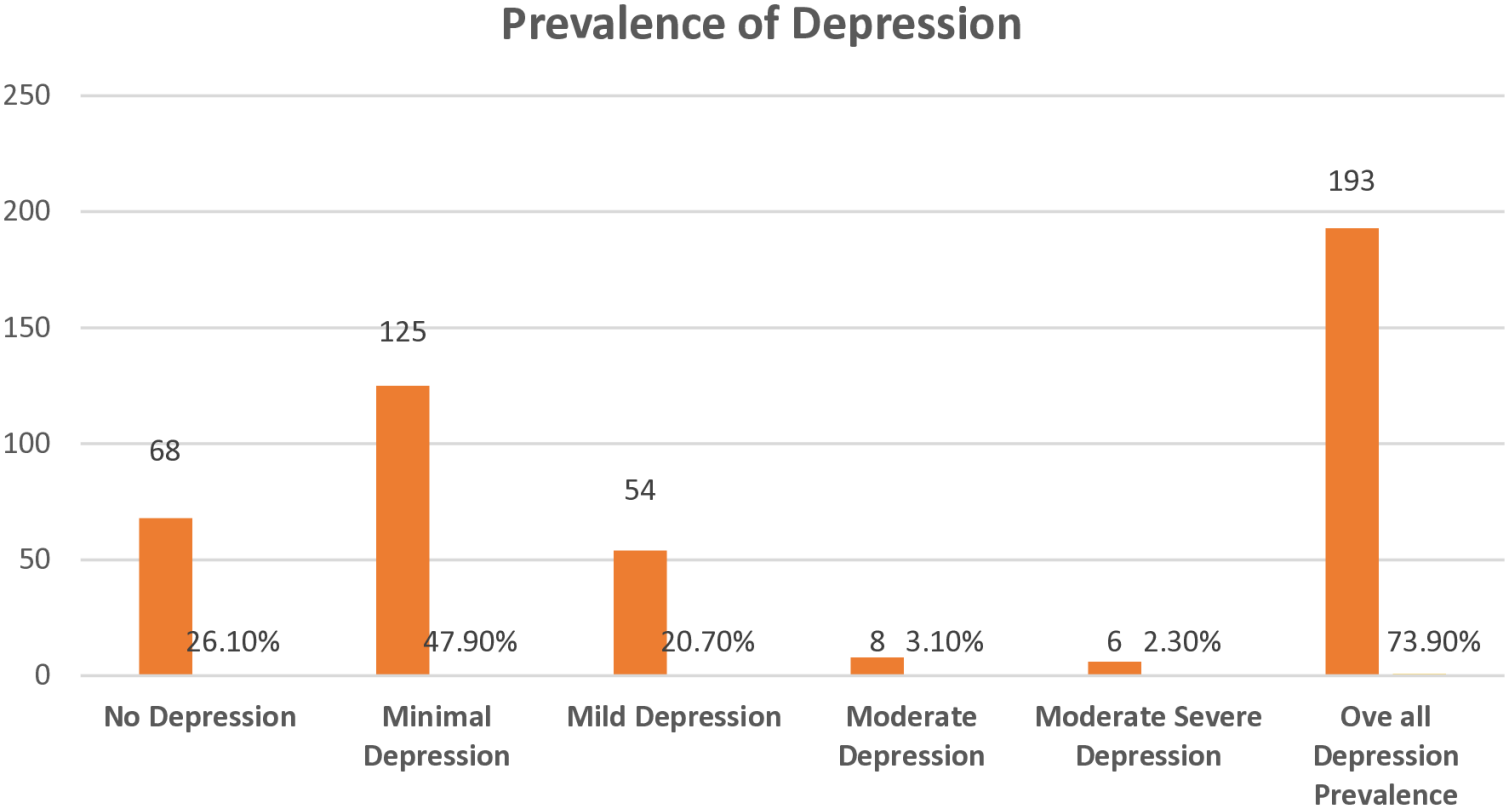
Prevalence of depression among respondents.

The study showed that 73.9% (n = 193) (95% CI 68.6-79.7) of respondents had depression. None of the respondents were found to have severe depression (**Fig 1).**

### Socio-demographic characteristics associated with depression among clients attending methadone clinics

Females had a 15 times increased risk of experiencing depression compared to males (adOR) of 15.126 (95% CI: 1.5-151.4; P = 0.021). Clients with a history of incarceration showed a two times increased risk for depression (adOR of 2.3 (95% CI: 1.2-4.6; P = 0.012)), and those incarcerated more than once have an even higher increased risk of depression (adOR of 4.066 (95% CI: 1.5-10.6; P = 0.004)). Other factors, such as age, marital status, education level, and employment status, were not significantly associated with depression (**Table 3**).

**Table 3:** Socio-demographic characteristics associated with depression among clients attending methadone clinics.

Variable	Category	Bivariate analysis				Multivariate			
		unOR	95% CI		P value	adOR	95% CI		P value
			Lower	Upper			Lower	Upper	
**Age (in years)**	18-24	Ref				Ref			
	25-34	0.085	0.008	0.893	0.040	0.091	0.007	1.103	0.060
	35-44	0.144	0.015	1.428	0.098	0.164	0.014	1.870	0.145
	45 and above	0.088	0.009	0.905	0.041	0.095	0.008	1.149	0.064
**Gender**	Male	Ref				Ref			
	Female	12.00	1.317	109.333	0.028	**15.126**	**1.511**	**151.456**	**0.021**
**Marital status**	single	Ref				Ref			
	married	1.172	0.613	2.241	0.632				
	Divorced	1.562	0.676	3.614	0.297				
**Level of education**	Primary	Ref				Ref			.
	Secondary	0.696	0.324	1.497	0.354	0.880	0.386	2.005	0.761
	Tertiary	0.476	0.056	4.053	0.497	0.973	0.109	8.664	0.981
	Illiteracy	2.854	1.070	7.610	0.036	2.269	0.766	6.726	0.139
**Employment status**	Employed	Ref				Ref			
	Unemployed	1.319	0.380	4.577	0.663	1.476	0.377	5.783	0.577
	Business owner	0.412	.149	1.138	0.087	0.590	0.189	1.844	0.364
**Incarceration**	Never					Ref			
	Once	2.529	1.366	4.683	0.003	2.371	1.212	4.639	0.012
	More than once	4.253	1.756	10.301	0.001	4.066	1.545	10.699	0.004

### Clinical characteristics associated with depression among clients attending methadone clinics

Individuals with HIV as a result of substance use were less likely to have depression (adOR = 0.019, 95% CI: 0.001-0.339; P = 0.007). Also, respondents who contracted TB as a result of substance use had a reduced risk of depression (adOR = 0.040, 95% CI: 0.006-0.286, P = 0.001), similar to those who reported no physical effects of using a substance (adOR = 0.081, 95% CI: 0.017-0.386, P = 0.002). Not using diclopar was associated with a reduced risk (adOR = 0.280, 95% CI: 0.136-0.576, P = 0.001), while not using tramadol was associated with a reduced risk of depression (adOR = 0.853, 95% CI: 0.121-0.530, P < 0.001). Having no one to share joy or sorrow was associated with an increased risk for depression (aOR = 4.606, 95% CI: 1.182-17.953; P = 0.028). Other factors, such as duration in the methadone programme, use of morphine/heroin, diazepam, tobacco, alcohol, cannabis, support during stress, and family history of depression or mental illness, were not statistically significant (**Table 4).**

**Table 4:** Clinical characteristics associated with depression among clients attending MAT Clinics.

Variable	Category	Bivariate analysis				Multivariate analysis			
		unOR	95% CI		Pvalue	adOR	95% CI		P value
**Duration in Methanone Treatment program (in months)**	3-9	Ref							
	10- 16	1.3	0.6	2.8	0.49				
	17-23	0.7	0.3	1.9	0.51				
	>24	0.9	0.4	1.8	0.68				
**The physical effect of substance use**	Injuries	Ref				Ref			
	HIV	0.1	0.04	0.6	0.054	**0.019**	**0.001**	**0.339**	**0.007**
	HBV	0.4	0.01	2.9	0.375	0.345	0.032	3.739	0.381
	TB	0.1	0.01	0.4	0.075	**0.040**	**0.006**	**0.286**	**0.001**
	None	0.12	0.02	0.4	0.122	0.081	0.017	0.386	0.002
**Used of diclopar**	Yes	Ref				Ref			
	No	0.4	0.2	0.8	0.005	**.280**	**0.136**	**0.576**	**0.001**
**Used tramadol**	Yes	Ref				Ref			
	No	2.3	0.2	0.6	0.000	**1.853**	**1.121**	**9.530**	**0.00**
**Used morphine/Heroine**	Yes	Ref							
	No	2.9	0.34	23.602	0.320				
**Diazepam**	Yes	Ref							
	No	0.751	0.412	1.369	0.350				
	No	0.415	0.154	1.114	0.081				
**Used Tobacco**	Yes	Ref				Ref			
	No	0.537	0.226	1.275	0.159	0.878	0.307	2.513	0.808
**Used of alcohol**	Yes								
	No	0.807	0.450	1.448	0.472				
**Used of cannabis**	Yes								
	No	0.688	0.356	1.328	0.265				
**Support during stress**	Yes	Ref							
	No	1.951	1.050	3.625	0.035				
**A person to share information during stress**	Family	Ref				Ref			
	Friends	2.288	0.891	5.875	0.085	1.999	0.548	7.293	0.294
	Other special person	3.861	1.447	10.306	0.007	2.354	0.501	11.058	0.278
	None	2.452	1.249	4.811	0.009	0.653	0.172	2.471	0.530
**Person to share with joy or sorrow**	Family	Ref				Ref			
	Friends	2.455	1.088	5.539	0.031	2.396	0.718	7.987	0.155
	Anyone special	2.382	.963	5.894	0.060	2.986	0.563	15.839	0.199
	none	3.054	1.498	6.224	0.002	**4.606**	**1.182**	**17.953**	**0.028**
**Being loved by**	Family	Ref				Ref			
	Friends	1.049	0.461	2.384	0.910	0.618	0.216	1.772	0.371
	Special	0.786	0.161	3.830	0.766	0.332	0.038	2.909	0.319
	none	4.045	1.430	11.440	0.008	2.607	0.724	9.387	0.143
**Family history of depression or mental illness**	Yes	Ref				Ref			
	No	0.303	0.148	0.621	0.001	0.462	0.190	1.125	0.089

## Discussion

This study aimed to investigate the prevalence and factors contributing to depression in individuals receiving methadone treatment. In this study, the prevalence of depression is high (73.9%). Gender and history of incarceration are the socio-demographic characteristics associated with increased risk of depression among clients in the study areas. Similarly, having no one to share joy or sorrow is the clinical factor associated with the increased risk of depression in the study population. In contrast, those having HIV or TB as a result of substance use, reporting no physical effects as a result of substance use and not using tramadol have a reduced risk of depression.

In this study, the overall prevalence of depression among respondents was about 12 times higher compared to the general population at 5.8% [27]. In this population, the vulnerability of depression may be due to rising inequality, limited social support, and a lack of permanent residents [28,29]. Furthermore, the findings in this study show that the social environment in which the study population lives contributes to the high prevalence of depression. This argument is supported by Ndayongeje et al. [30], who revealed that people who use drugs live in a lonely environment with limited social support. A study in Malaysia reported a lower prevalence (13.8%) of depression [13] than that we found in our study, suggesting that variations in healthcare systems in the methadone clinic play a role in the reduced risk of depression between the two countries. It is worthwhile to note that our findings contradict those reported in the systematic review, which shows that using methadone is associated with reduced depression [31]. The current study was a cross-sectional study, which cannot be used to describe associations, as opposed to the cited review, which may have included prospective studies that may have followed up clients over time. However, the role of methadone in reducing depression is an interesting area that needs further investigation.

Our study found a higher risk of depression among females, consistent with global research [32–34]. For females who use opioids, the roles of women as wives, employees, mothers, and carers contribute to their daily stress levels, leading to increased depression levels. In addition, evidence shows that the experience of stigma, economic disadvantage and having less social support increases the risk of depression among females [35,36]. This is also similarly reported in Nigeria, where females in substance use treatment often face additional stressors such as caregiving responsibilities, social stigma, and higher rates of trauma and violence, contributing to higher depression rates. This emphasises the need for gender-sensitive approaches in treatment programmes [37]. The current study did not find a significant association between age or marital status and depression, which contrasts with a study reported in Nepal suggesting that younger individuals and those who are single or divorced may be more susceptible to depression [38]. This difference might be due to specific cultural or social support structures in our study population.

A history of incarceration significantly increases the risk of depression in our study. This can be described by the fact that the prison or cell environment can exacerbate depressive symptoms. A systematic review revealed that prisoners are more likely to have a depressive disorder than the general population [39]. Poor living conditions, such as lack of personal freedom, feeling bored and losing a sense of purpose, might contribute to the depressive symptoms as evidenced in our study. This finding is in line with that reported in South Africa [40], which showed that the trauma and stress associated with incarceration contribute to mental health issues. The higher depression rates among previously incarcerated individuals highlight the need for post-incarceration support programmes to address mental health needs.

Respondents with HIV or TB had a lower risk of depression compared to those with substance use-related injuries. Methadone treatment services offer a strategic approach to lowering illness and death rates related to TB and HIV among high-risk drug users and their communities, focusing on delivering comprehensive, person-centred care [41,42]. This might have contributed to reducing depression risk in our study population. This finding may seem counterintuitive but could be explained by better integration of healthcare services and support systems for individuals with chronic conditions such as HIV or TB, which has been intensively implemented in Tanzania. Methadone treatment has been shown to improve the treatment of TB among hospitalised patients in Ukraine [43] and the United States of America (USA) [44,45]. Our study provides insight into the combined effect of depressive symptoms among individuals with HIV, TB and substance use. This warrants more intervention research to find effective strategies for the reduction of depressive symptoms among the study populations.

Our study found that clients who were not using diclopar and tramadol were associated with a lower risk of depression. Tramadol, as an opioid drug, affects the pain receptors in the brain. This acts to block pain, as well as boosting feelings of pleasure [46–48]. When Tramadol and Diclopar are abused or misused, they can create a euphoric state which, on chronic use, can lead to symptoms of depression [49]. Therefore, avoiding the use of tramadol or diclopar may reduce depressive symptoms as reported in the findings of our study. This aligns with findings by Cui et al. [50] in Canada and by Biggar et al. [51] in the USA, who reported that the use of certain substances, especially opioids and other depressants, is linked to higher depression rates. This suggests that methadone programmes should closely monitor the use of these substances and provide appropriate mental health support in all methadone clinics in Tanzania.

The significant role of social support in mitigating depression is evident in our study, with those lacking social support having a markedly higher risk of depression. This finding is supported by Dai & Smith [15], who emphasised that robust social support networks are crucial for improving mental health outcomes. Our results underscore the importance of integrating social support services into methadone treatment programmes.

### Strengths and limitations

The strengths of this study included the use of validated depression scales. The PHQ-9 has been validated and used to collect data related to depression for various populations in Tanzania. However, the study should be interpreted in light of some important limitations. First, respondents may have had difficulty accurately recalling past experiences or symptoms, leading to potential recall bias. This was mitigated by using a tool to assess depression, which had clear and specific time frames that helped minimise recall bias. Additionally, this was a cross-sectional study where exposure and outcome were measured simultaneously. Therefore, determining cause and effect in this study was challenging. However, the study identified key factors that could be useful for clinical intervention in the study population.

## Conclusion

The high prevalence of depression among individuals undergoing methadone treatment is associated with gender, history of incarceration, and the presence of physical illnesses such as TB/HIV. Also, social support is crucial in curbing and improving depressive symptoms in the study population. These findings underscore the need for targeted and comprehensive MAT services that address the needs of those addicted to opioids in Tanzania.
